# A 55‐year‐old COVID‐19‐positive man managed with self‐regulation of high‐flow oxygen by high‐velocity nasal insufflation therapy

**DOI:** 10.1002/rcr2.591

**Published:** 2020-05-21

**Authors:** Ari J. Ciment, Lawrence M. Ciment

**Affiliations:** ^1^ Mount Sinai Medical Center Miami Beach FL USA

**Keywords:** COVID‐19, nasal insufflation, patient control, PPE, Vapotherm

## Abstract

Management of critically ill coronavirus disease 2019 (COVID‐19) patients remains both risky and technically challenging. A 55‐year‐old male COVID‐19‐positive patient with obstructive sleep apnoea (OSA), diabetes, and obesity presented with cough and shortness of breath, escalating to requiring high‐flow oxygen therapy by high‐velocity nasal insufflation. The patient's flow rate and oxygen fraction remained labile throughout much of the hospitalization. This lability required frequent clinician interactions and use of personal protective equipment. The patient was alert and oriented and was instructed on the operation of the high‐flow system, specifically the adjustment of both flow rate and oxygen percentage. The patient was instructed to modify oxygen to maintain an SpO_2_ (peripheral capillary oxygen saturation) target range, and flow rate to address dyspnoea as well as reduction of flow as tolerated when other staff entered the room. The patient was successfully self‐regulated for 10 days and was discharged on 2 L/min nasal cannula on day 14 of his illness.

## Graphical abstract

We present a case report on a 55‐year‐old male with obstructive sleep apnoea (OSA), diabetes, and obesity requiring escalation to high‐flow oxygen by high‐velocity nasal insufflation (HVNI; Vapotherm). As this patient required frequent clinician interaction and use of personal protective equipment (PPE) to control flow rate and oxygen fraction due to lability, and the patient was alert, we instructed the patient to modify the oxygen to maintain an SpO_2_ (peripheral capillary oxygen saturation) target range and flow rate, to address dyspnoea as well as reduction of flow, as tolerated, when other staff entered the room. This was a successful 10‐day self‐regulation of HVNI, wherein on day 14 the patient was discharged.

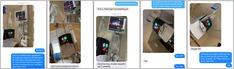

## Introduction

Critically ill patients with coronavirus disease 2019 (COVID‐19) pneumonia often require oxygenation support to maintain oxygen saturation target levels [1]. Current guidelines include the use of high‐flow oxygen therapy for such intervention [1, 2]. High‐velocity nasal insufflation (HVNI) includes the ability to provide high‐flow nasal oxygen [3]. All high‐flow therapies carry a requirement for close monitoring and adjustment, as well as the potential for aerosol generation; both concerning to clinical staff.

## Case Report

**Figure 1:**
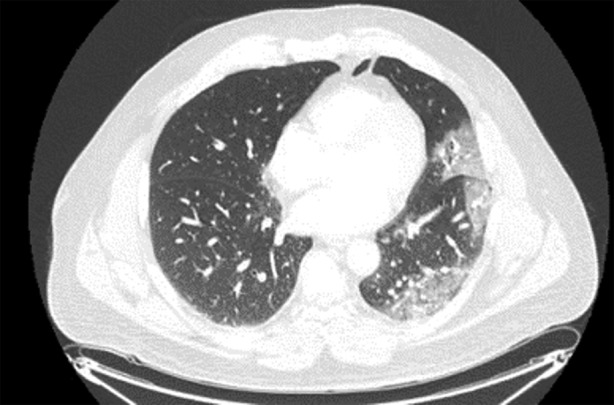
Chest non‐contrast axial computed tomography (CT) scan, note ground‐glass opacity of the left lung.

A 55‐year‐old man with obesity, obstructive sleep apnoea (OSA), and diabetes mellitus presented with cough and shortness of breath for seven days and oxygen saturation of 88% on room air. Laboratories were significant for lymphopenia and high neutrophil‐to‐lymphocyte ratio of 3.6. He was initiated on treatment with hydroxychloroquine, zinc, and azithromycin (Zithromax, Pfizer, USA) but his oxygen saturation and dyspnoea worsened over the next three days. Chest computed tomography (CT) was consistent with COVID‐19 (Fig. 1). His airway was a Mallampati score 4 and despite desaturating to 87% on 8 L/min nasal cannula, it was decided to give him a trial of HVNI (Precision Flow Hi‐VNI; Vapotherm, Inc., USA) via nasal cannula, under a surgical mask (to reduce aerosol dispersal) [4].

The primary objective was to provide effective treatment of the patient whilst using this novel approach, allowing the patient to assist with effective self‐monitoring and adjustment, while maintaining direct contact with the attending physician, after education and understanding the disease process and training on the limited control of the equipment. The patient understood that this would also subsequently minimize patient–clinician interactions to protect clinical staff from avoidable exposure risk. The patient was instructed on the operation of the HVNI high‐flow device, on adjustment of oxygen fraction (FiO_2_) to achieve an SpO_2_ (peripheral capillary oxygen saturation) target value, and on adjustment of flow rate for both dyspnoea relief and to reduce exposure to staff when they entered the room (reduction in flow as tolerated by the patient). This patient activity constituted self‐regulation. This was made possible by the patient's facility using his smartphone, specifically FaceTime audio/video chat and SMS/picture messaging. The smartphone permitted high‐frequency interaction with a clinician outside the room to assess patient decision‐making regarding both FiO_2_ and flow rate adjustments. The patient was able to demonstrate successful decision‐making during training and in subsequent follow‐up. Self‐adjustment by the patient was endorsed by the managing clinician after assessment by message‐based interactions.

**Figure 2:**
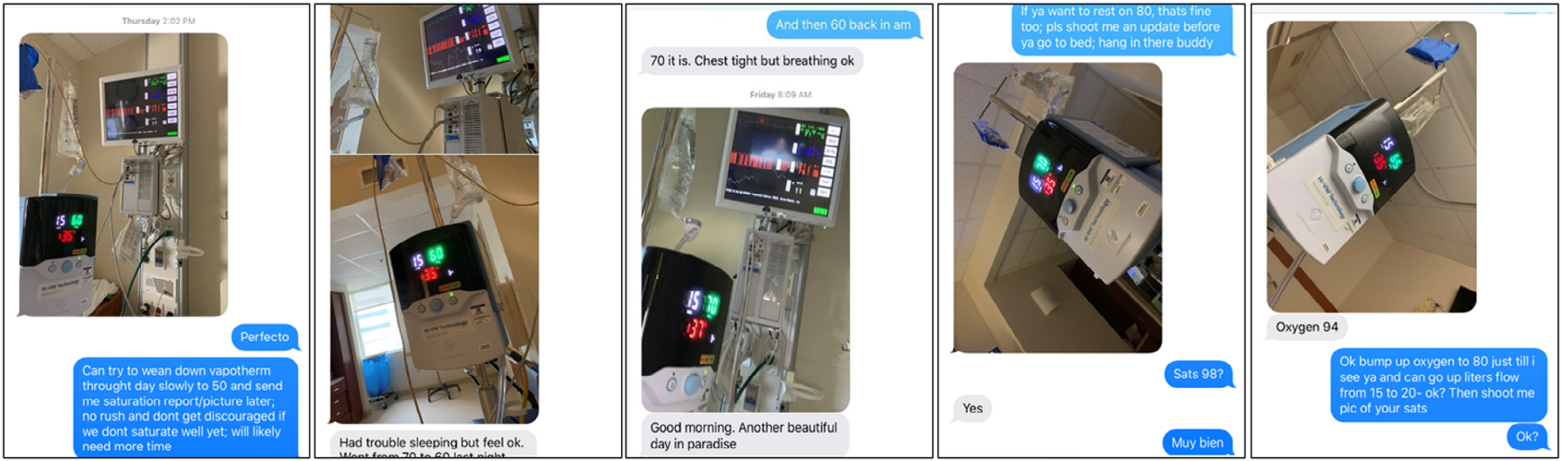
Typical messaging interaction with image capturing both high‐flow settings and simultaneous monitor readout. Note the interactive character of the interaction, patient addition of status, and subsequent instructions (clinician communication in blue).

This self‐adjustment facilitated a sense of personal engagement by the patient, with the patient using electronic messaging sent to the attending physician for assessment. Patient self‐adjustment continued for 10 days, with FaceTime and message‐based interaction with the attending physician at a minimum of approximately every 1–2 h (Fig. 2). Adjustments were appropriate, and this self‐adjustment continued despite his rising C‐reactive protein (CRP, up to 81 mg/L) and shortness of breath peaking on illness day 10. The patient was able to ride out the cytokine storm and acute respiratory distress syndrome (ARDS) while performing these adjustments. This patient control also reduced the overall requirement for the use of personal protective equipment (PPE) as staff would have otherwise had to enter and leave the room regularly to perform such adjustments.

The patient was discharged on 2 L/min oxygen and feeling well on day 14 of his illness. No healthcare workers in contact with the patient became infected with COVID‐19 during these procedures.

## Discussion

Management of type I respiratory failure associated with viral pneumonia has become an important focus, after the epidemic of severe acute respiratory syndrome and Middle Eastern respiratory syndrome. Post‐epidemic analysis has included evaluation not only of the efficacy of various therapeutic modalities, but also of potential risk to staff/patients for nosocomial risk associated with such therapies. Intubation and mechanical ventilation are required for management of severe ARDS but is not without challenges. Intubation presents the most profound challenge to the clinician in terms of potential risk of exposure to aerosolized particles, and mechanical ventilation becomes a limited resource during a pandemic event such as COVID‐19. Non‐invasive approaches are important considerations to help reduce those burdens. The management of these patients using non‐invasive approaches calls for close clinical observation and adjustment of therapy, as necessary.

The ability to self‐regulate the therapy was successful for this patient due to his level of consciousness and his facility with the smartphone applications. This will almost certainly not be reproducible across all patients. However, a sizable proportion of patients with the low elastance phenotype of COVID‐19 may be candidates not only for high flow oxygen (HFO) therapy, but also for self‐regulation [5]. The staff taking care of this patient reported significantly less use of PPE as well as less frequent entry into the patient room. Standard monitoring continued without pause during his care. The use of this method of management seems to provide a novel mechanism to provide essentially telehealth services from an intensive care environment. Further study may be warranted.

### Disclosure Statement

Appropriate written informed consent was obtained for publication of this case report and accompanying images.

At the time this report was accepted for publication, the authors declared that this patient had not been included in any previously published report on COVID‐19 that they had authored.
